# Assessing psychological distress in cancer patients in intensive care: Validation of the intensive care psychological assessment tool in Mexico

**DOI:** 10.1017/S1478951525100667

**Published:** 2025-08-28

**Authors:** Mariana Flores-Constantino, Francisco Lorenzo Juárez-García, Adriana García-García, Tecelli Dominguez-Martínez, Abigail Quintana-Sánchez, Nazira Calleja-Bello, Oscar Galindo-Vázquez, Manuel S. Ortiz, Tania Estapé, Silvio A. Ñamendys-Silva

**Affiliations:** 1 Facultad de Psicología, Universidad Nacional Autónoma de México (UNAM), Mexico City, México; 2 Investigaciones Epidemiológicas y Psicosociales, Instituto Nacional de Psiquiatría “Ramón de la Fuente Muñiz”, Mexico City, México; 3 Unidad de Cuidados Intensivos, Instituto Nacional de Cancerología, Mexico City, México; 4 Centro de Investigación en Salud Mental Global, Instituto Nacional de Psiquiatría “Ramón de la Fuente Muñiz”, Mexico City, México; 5 Jefatura de Enfermería, Instituto Nacional de Cancerología, Mexico City, México; 6 Departamento de Salud Mental, Instituto Nacional de Cancerología, Mexico City, México; 7 Dirección del Programa de Doctorado en Psicología, Universidad de la Frontera, Temuco, Chile; 8 Departamento de Psicooncología, FEFOC Fundación Barcelona, Barcelona, Spain

**Keywords:** ICU, cancer, IPAT, psychometric properties, Mexican population

## Abstract

**Background:**

Individuals admitted to the Intensive Care Unit (ICU) due to cancer frequently encounter cognitive impairment and alterations in their mental health, which engenders psychological distress and considerably impacts their quality of life. In Mexico, there is an imperative for valid and reliable clinical tools to identify these symptoms, to providing timely and appropriate psychological intervention.

**Objectives:**

To determine the psychometric properties of the Intensive Care Psychological Assessment Tool (IPAT) in a Mexican population with cancer discharged from ICU.

**Methods:**

A cross-sectional instrumental design with non-probability convenience sampling was employed. Data were collected between February 2023 and October 2024 with 75 people discharged from the ICU. Factor structure (confirmatory factor analysis), reliability (internal consistency), measurement invariance, and criteria validity (convergent, discriminant, and known-groups) were assessed. Patients were assessed during oncological hospitalization, following ICU.

**Results:**

The participants were predominantly male, residing in the interior of the country, with an average age of 44 years (range 19–78, SD 16.21). Internal consistency results were deemed to be satisfactory (α = 0.78) for 9 items. The CFA indices were adequate [χ^2^ (gl) 27.436 (24), CMIN/DF 1.143, CFI 0.96, GFI 0.97, SRMR 0.036, RMSEA 0.044] as were the scalar invariance indices for invasive mechanical ventilation [CFI = 0.871; RMSEA = 0.058; χ^2^/gl = 20.519 (10)] and for gender, restricted invariance indices [CFI = 0.849; RMSEA = 0.068; χ^2^/gl = 23.302 (12)].

**Significance of results:**

The Mexican version of the IPAT for people with cancer is a valid and reliable tool for use in oncology and critical care settings in Mexico. It is recommended for use at the time of discharge from the ICU, as it allows the identification of psychological distress for timely intervention. For future considerations, diverse clinical settings and patient populations should be explored to enhance the tool’s applicability and generalizability in the varied contexts of cancer in ICU.

## Introduction

Cancer is a problem worldwide public health problem (Zamudio-Sánchez et al. 2022), and mental health issues affecting patients throughout their cancer journey are becoming increasingly relevant. Symptoms of anxiety and depression are prevalent among cancer patients (Pitman et al. 2018; Unseld et al. 2021). In Mexico, symptoms of depression have been reported in 24–40% of the cancer population, and symptoms of anxiety in 29–58.8% (Cu-Menes et al. 2020; Hernández-Marín 2019).

This burden on mental health becomes even more pronounced when cancer patients are admitted to an Intensive Care Unit (ICU), is it to restore clinical stability for continued treatment and quality of life (Rigaud et al. 2017) but it can greatly intensify psychological distress due to severe clinical conditions and perceived threats to life. Evidence shows that the psychological impact of ICU stays can be severe (Wade et al. 2014). In Mexico, 200–300 cancer patients are admitted to the ICU annually (INCan, Instituto Nacional de Cancerología (INCan) 2024).

Research has revealed that more than 50% of individuals admitted to this unit exhibited symptoms of a psychological disorder following admission. Cognitive deficits in memory, attention, and executive function, which impact activities of daily living, are also prevalent symptoms in this population group (Pandharipande et al. 2017).

It has been demonstrated that psychological distress in the ICU constitutes one of the most significant risk factors for adverse psychological prognoses following critical care (Wade et al. 2012). Consequently, it is necessary to detect and mitigate this condition to the greatest extent possible. Nevertheless, challenges in recognizing this condition may be encountered by healthcare professionals, including the presence of delusions (Pouwer et al. 2006; Spronk et al. 2009).

A kind of instruments are employed in this field, including the 4P questionnaire (Åkerman et al. 2013), Beck Depression Inventory (Bienvenu and Ginsburg 1996), ICU memory tool (Jones et al. 2000), and PCL-5 (Bovin et al. 2015), among others (Creamer et al. 2003; Kessler et al. 2002; Kroenke et al. 2001; Rattray et al. 2004; Twigg et al. 2008; Zigmond and Snaith 1983), aimed at identifying various psychological disturbances, including physical and psychosocial problems, post-traumatic stress disorder, anxiety, and/or psychological distress.

Despite the high specificity and internal consistency exhibited by these scales, they are not specifically designed to assess individuals with cancer receiving intensive care.

Another limitation, within the context of the UCI, is unclear whether this event should be considered the reason for hospital admission or whether it refers to the procedures undergone within the hospital itself. In this complex situation, utilizing a specific measure of psychological well-being in the ICU would be more appropriate.

The Intensive Care Psychological Assessment Tool (IPAT) developed by Wade et al. (2014), is characterized by its clinical and methodological features, rendering it suitable for utilization in this context. This instrument is a straightforward and accessible 10-item scale with three response options that can be employed routinely.

It can be administered to patients who are either awake and alert or who have been roused. Furthermore, the scale can be completed by the healthcare team for timely detection and psychological care.

Additionally, the psychometric properties of the instrument have been demonstrated to be valid in both the original version in British English (Wade et al. 2014) and its subsequent validations. The instrument’s psychometric properties are adequate for the assessment of psychological distress.

Although Spanish versions of the instrument have been validated in other Latin American countries, it is essential to conduct a specific cultural and clinical adaptation for the Mexican context, because it has distinct sociocultural characteristics that may influence how people perceive, and express experiences related to illness, distress, and coping (Kleinman 1988).

Furthermore, they do not report the etiology of admission to the ICU, which hinders the determination of whether these studies are focused on the cancer population. Moreover, this adaptation targets individuals with cancer, a population with unique psychological and clinical needs, the emotional processes, existential concerns, and family dynamics involved in the context of cancer are specific and require accurate, culturally sensitive assessment (Holland et al. 2021)

Given these limitations, the present study aims to (1) determine the psychometric properties of the IPAT in Mexican patients with cancer following ICU, and (2) establish a reliable and valid scale that can effectively identify mental health problems in this demographic. This will allow for timely psychological interventions and contribute to improved quality of life.

## Method

### Participants

Non-probability convenience sampling was employed, with data obtained from hospitalization of Nacional Cancer Institute in México between February 2023 and October 2024. The design was cross-sectional instrumental (Montero and León 2005). The sample size was determined considering a minimum of ≥ 5 participants per item (Lloret-Segura et al. 2014), resulting in a total of ≥ 50 individuals. The participation criteria for this research were as follows:

- Inclusion: consent form signed, oncological diagnosis, ICU length of stay ≥ 48 h, discharged from the ICU, adults (≥18 years), Spanish as native language, adequate cognitive ability (oriented in time, space, and person) (Folstein et al. 1975; Lezak et al. 2012), Karnofsky Index score ≥ 50 (assessed by an oncologist).
- Exclusion: Psychological disorders of anxiety, depression, and/or post-traumatic stress disorder, diagnosed prior to ICU admission.
- Elimination: Chose not to participate or assessments not completed.

### Measurements

- Identification form: Developed to record sociodemographic and clinical data (age, sex, educational level, residence, current occupation, children, relationship status, cancer diagnosis, oncological treatment, comorbidities, reason ICU admission, and days in the ICU).
- Intensive Care Psychological Assessment Tool (IPAT) (Wade et al. 2014), designed to assess psychological distress in critical care settings, comprises 10 items, and its test–retest reliability has been determined to be 0.8. Concurrent validity with measures of anxiety and depression has shown to be *r* = 0.7, *P* < 0.01; *r* = 0.6, *P* < 0.01, respectively. The cut-off point for the detection of concurrent anxiety is ≥ 7, with a sensitivity of 82% and a specificity of 65%. For the detection of concurrent low mood, the cut-off point is also ≥ 7, with a sensitivity of 80% and a specificity of 66%.
- The Quality-of-Life scale (EORT-C QLQ C30), specific focus on cancer patients (Aaronson et al. 1993) and has been validated in the Mexican population by Oñate-Ocaña et al. (2009). The scale assesses five functional domains (physical, performance, cognitive, emotional, and social), three symptomatic domains (fatigue, pain, and nausea/vomiting), a global quality of life scale, and various symptoms frequently reported by cancer patients. Cronbach’s alphas range from 0.32 to 0.90. (Global QoL α = 0.90, Physical α = 0.79, Role α = 0.80, Emotional α = 0.85, Cognitive α = 0.32, and Social α = 0.80), which aligns with previous findings and is attributed to the limited number of items in each domain. Statistically significant concurrent validity (Pearson’s *r* from 0.37 to 0.43, *P* < 0.05) between the EORTC QLQ-C30 and albumin levels has been demonstrated.

## Procedure

### Adaptation process

First, two professional translators, fluent in both languages and familiar with construct and both cultures, independently translated, aiming to preserve the original meaning while using expressions natural to the target population. They were informed of the scale’s intent to ensure semantic and cultural accuracy (Reyes-Lagunes and García-Barragán 2008).

Once direct translation was available, the decision was made to choose the most appropriate version, considering (a) that the meaning and intention of the item was preserved, and (b) that the language was clear and natural for the target population.

Then, the best version translated was sent for back-translation by other independent bilingual translators, whose native language was that of the original version. In this step, the translators were not supposed to know the scale and its purpose (Muñiz et al. 2013).

The back-translated version was compared item by item with the original, evaluating the degree of equivalence through the judgment of bilingual translators and construct experts. The most appropriate wording for the target population was selected based on consensus (see Supplementary Material).

### Content validity

Subsequently, the scale was evaluated by 8 experts. It aimed at ascertaining 4 key areas: pertinence, clear relevance, and appropriate wording and language appropriate for the target population.

To evaluate the pertinence of the items, the Content Validity Ratio (CVR) (Lawshe 1975) was used, using a dichotomous scale (yes/no) (see Table 1). All items are essentially valid for measuring the proposed construct.

**Table 1.** Content validity ratio for the IPAT by 8 experts

	Pertinence of the items								
	*Experts*								
	*Yes = 1, No = 0*								
*Items*	*1*	*2*	*3*	*4*	*5*	*6*	*7*	*8*	*CVR*
1. ¿Ha sido difícil comunicarse?	1	1	1	1	1	1	1	1	1.00
2. ¿Ha sido difícil dormir?	1	1	1	1	1	1	1	1	1.00
3. ¿Se ha sentido tenso/a?	1	1	1	1	1	1	1	1	1.00
4. ¿Se ha sentido triste?	1	1	1	1	1	1	1	1	1.00
5. ¿Ha sentido pánico?	1	1	1	1	1	1	1	1	1.00
6. ¿Se ha sentido sin esperanza?	1	1	1	1	1	1	1	1	1.00
7. ¿Se ha sentido desorientado/a (no muy seguro/a de dónde se encuentra)?	1	1	1	1	1	1	1	1	1.00
8. ¿Ha tenido alucinaciones (escuchó o vio cosas que sospechaba que en realidad no existían)?	1	1	1	1	1	1	1	1	1.00
9. ¿Ha sentido que las personas estaban tratando deliberadamente de lastimarle o herirle?	1	1	1	1	1	1	1	1	1.00
10. ¿Siguen viniendo a su mente recuerdos angustiantes de su estancia en Cuidados Intensivos?	1	1	1	1	1	1	1	1	1.00

Additionally, to evaluate more specific aspects of item quality, Aiken’s V (Aiken 1980) was used (see Table 2).

**Table 2.** Aiken’s V of the IPAT by 8 experts

	Relevance of the items									
	*Experts*									
	1 = not relevant to									
	5 = very relevant and clear									
*Items*	1	2	3	4	5	6	7	8	*Mean*	*Aiken’s V (IC95%)*
1. ¿Ha sido difícil comunicarse?	5	5	5	5	5	5	5	5	**5**	*1.00(.89 − 1.00)*
2. ¿Ha sido difícil dormir?	4	5	5	5	5	5	5	5	**4.8**	*0.97*
										*(0.84–0.99)*
3. ¿Se ha sentido tenso/a?	5	5	5	5	4	5	5	5	**4.8**	*0.97*
										*(0.84–0.99)*
4. ¿Se ha sentido triste?	5	5	5	5	5	5	5	5	**5**	*1.00*
										*(0.89−1.00)*
5. ¿Ha sentido pánico?	5	5	5	5	5	5	5	5	***5***	*1.00*
										*(.89 − 1.00)*
6. ¿Se ha sentido sin esperanza?	5	5	5	5	5	5	5	5	***5***	*1.00*
										*(0.89−1.00)*
7. ¿Se ha sentido desorientado/a (no muy seguro/a de dónde se encuentra)?	5	5	5	4	4	5	5	5	***4.75***	*0.94*
										*(0.80–0.98)*
8. ¿Ha tenido alucinaciones (escuchó o vio cosas que sospechaba que en realidad no existían)?	4	5	5	4	5	5	5	5	***4.75***	*0.94*
										*(0.80–0.98)*
9. ¿Ha sentido que las personas estaban tratando deliberadamente de lastimarle o herirle?	5	5	5	5	5	5	4	4	**4.75**	*0.94*
										*(0.80–0.98)*
10. ¿Siguen viniendo a su mente recuerdos angustiantes de su estancia en Cuidados Intensivos?	4	5	5	4	4	5	4	4	**4.37**	*0.84*
										*(0.68–0.93)*

All items had meaning equal to or above 4.37 and Aiken´s V values between 0.84 and 1.00, indicating a high rating for relevance and clarity.

Judges agreed that the items are clearly and grammatically well-structured, with vocabulary appropriate to the target population’s cognitive, cultural, and linguistic context, which guarantees a correct interpretation of the content.

These strongly support the content validity of the instrument, items are relevant, meaningful and understandable.

### Pilot

It was conducted with 10 patients who met the inclusion criteria and were asked if they had difficulty answering the items, if it was confusing or contained difficult words, if it was offensive and if they would rephrase the item (Díaz-Muñoz 2020). There was consensus in their responses and no modifications were made.

### Application

Following ICU, patients were transferred to the hospital wards, where the recruitment process took place. An ICU nurse identified potential participants and psycho-oncologist responsible for evaluating patients, verify that they met inclusion criteria. This procedure was the same for all patients and was administered during their post-ICU hospital stay.

Table 3 details the number of days between discharge from the ICU and the assessment, with an average of 4 days and a range of 1–30 days.

**Table 3.** Sociodemographic and clinical characteristics of a sample of 75 participants with cancer discharged from ICU

Age in years: X̄ = 44, SD = 16.21, range = 19−78					
Variable	*f*	%	Variable	*f*	%
**Gender**			**Cancer diagnosis**		
Woman	36	48	Gastrointestinal	16	21.3
Man	38	50.7	Genitourinary	9	12
Non-binary	1	1.6	Gynecologic	17	22.7
**Children**			Hematologic	23	30.7
Yes	50	66.7	Breast	1	1.3
No	25	33.3	Musculoskeletal	1	1.3
**Residence**			Head and neck	2	3.3
Mexico City	24	34	Thoracic	3	4
State of Mexico	24	32	Skin	2	2.7
Interior of the Republic	25	33.3	**Stage (TNM)**		
**Education**			I	6	8
None	1	1.3	II	9	12
Primary education	18	24	III	13	17.3
Secondary education (GCSE)	19	25.3	IV	27	36
A-levels/BTEC -Foundation Degree/HND	26	34.7	Not applicable	6	8
Bachelor’s degree	9	12	Unknown	13	17.3
Postgraduate	1	1.3	**Cancer treatment**		
Special Educational Needs (SEN)	1	1.3	Chemotherapy	28	37.3
**Marital status**			Radiotherapy	4	5.3
Single	33	44	Surgery	16	21.3
Married	20	26.7	Surgery, chemotherapy and radiotherapy	5	6.7
Free union	14	18.7	No treatment	7	9.3
Widowed	5	6.7	Other	11	14.6
**Occupation**			**Comorbidity**		
Homemaker	17	22.7	Yes	25	33
Employee	17	22.7	No	50	66.7
Trader	9	12	**Mental healthcare throughout the lifespan**		
Professional	3	4	Yes	20	26.7
Unemployed	22	29.3	No	55	73.3
Student	1	1.3	**Type of mental healthcare**		
Deprived of liberty	2	2.7	Psychology	16	21.3
**Admission to ICU**			Psychiatry	0	0
Septic shock	37	49.3	Both (Psychiatry/ Psychology)	1	1.3
Hypovolemic shock	8	10.7	Other	3	4
Mixed shock	11	14.7	**Days in the ICU**		
Respiratory failure	4	5.3	≥ 2 days	50	66.7
Renal failure	11	14.7	7 days	9	12
Heart failure	2	2.7	> 7 days	15	20
**Invasive mechanical ventilation (IVM)**			**N° ICU procedures**: X̄ = 3, SD = 1.36, range 1−6		
Yes	34	45.3	**Days between assessment and discharge from ICU**: X̄ = 4, SD = 4.35, range = 1–30		
No	41	54.7	**Karnofsky Index after ICU:** X̄ = 71.96, SD = 14.13, range = 40−100		

Instruments were administered verbally, with patients who met the criteria: identification form, IPAT, and EORTC QLQ-C30.

## Data analysis

Analyses were conducted using SPSS version 26, with a significance level of *P* < 0.05 being set. This also entails verifying that the Kaiser–Meyer–Olkin (KMO) sample adequacy indices and Bartlett’s tests of sphericity were satisfactory for the subsequent analyses.

Reliability was obtained through internal consistency (Cronbach’s alpha) and validity of factor structure, explained by a confirmatory factor analysis using AMOS 22.

The confirmatory model was assessed using indices such as the chi-squared statistic (χ^2^), the chi-squared statistic divided by the degrees of freedom (χ^2^/gl), goodness-of-fit indices (GFI, NFI), comparative fit indices (CFI), and root mean square error of approximation (RMSEA) (Byrne 2010; Ullman 2006). Multigroup factor analysis was used to analyze measurement invariance with respect to sex and mechanical ventilation support. Finally, to substantiate criterion validity (convergent and discriminant), Pearson’s correlations were employed with a significance level of *P* < 0.05, also Known-Groups Validity was analyzed using Mann–Whitney U-test and Standarized Mean Differences (SMD), contrasting three variables by comorbidity, active oncologic treatment and invasive mechanical ventilation, within two groups each one.

## Results

### Clinical and sociodemographic characteristics

The 75 subjects who consented to participate are delineated in Table 1. Data reveal that 50.7% of the participants identified as male, with an average age of 44 years (SD = 16.21, range = 19–78).

### Items description

The results obtained from the items are presented in Table 4, including alpha if item deleted, mean, standard deviation (SD), range, frequency, floor or ceiling effects, skewness, kurtosis, inter-item correlation, corrected homogeneity index (cHI), extreme groups, and mean and SD total scale. Item 9 was eliminated based on alpha if item deleted (α = 0.78), SD (0.358), frequency distribution (>90% in 1 response option), skewness and kurtosis (>1), item–item correlation (<0.20), corrected homogeneity index (>0.30), and communalities (<0.50).

**Table 4.** Descriptive evaluation of the IPAT items

Total scale: Mean = 14.34, SD = 4.04										
Item	Alpha if item deleted	Mean	Standard deviation	Range	Frequency	Floor or ceiling effects^a^	Skewness/ Kurtosis^a^	Inter-item Corr.	Corrected homogeneity index (cHI)	Extreme groups
1	0.76	1.56	0.820	1−3	65.3	65.6/20.8	0.997/−0.756	0.18/0.50	0.44	.000
2	0.76	1.97	0.787	1−3	38.9	31.9/29.2	.049/−1.371	0.10/0.50	0.46	0.000
3	0.75	1.75	0.755	1−3	38.9	41.7/19.4	0.396/ −1.134	.09/.48	0.49	0.000
4	0.75	1.68	0.747	1−3	48.6	48.6/16.7	0.602/−0.962	.03/0.44	0.44	0.000
5	0.73	1.47	0.750	1−3	68.1	68.1/15.3	1.230/−0.066	0.16/0.55	0.57	0.000
6	0.75	1.31	0.597	1−3	76.4	76.4/6.9	1.826/2.248	0.01/0.55	0.45	0.000
7	0.77	1.39	0.662	1−3	70.8	70.8/9.7	1.472/0.883	−0.06/0.34	0.32	0.003
8	0.75	1.49	0.712	1−3	63.9	63.9/12.5	1.134/−0.087	0.27/0.41	0.51	0.000
9	0.78	1.11	0.358	1−3	90.3	90.3/1.4	3.42/12.213	−0.06/0.28	0.25	0.008
10	0.77	1.49	0.712	1−3	63.9	63.9/12.5	1.134/−0.087	−0.02/0.41	0.37	0.000

corr. = correlation,

a = percentage.

KMO = 0.712 and Bartlett’s test of sphericity: (*χ*^2^(36) = 155.782 *P* < 0.0001) indicate that the sample size was adequate for the subsequent analyses.

### Internal consistency

Obtained by Cronbach’s alpha, it was 0.78 for 9 items (Cronbach 1951; Furr 2018; John and Benet-Martinez 2000).

### Confirmatory factor analysis

**Figure 1.**
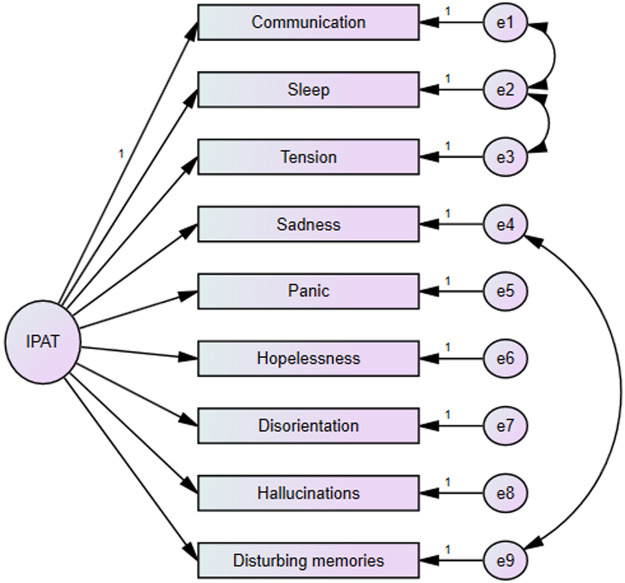
Final model of the Confirmatory Factor Analysis IPAT.

This analysis was undertaken to ascertain the scale structure documented in the various iterations of the scale. The modification indices indicated the necessity to establish covariances between the residuals, which were explored through different covariance models detailed in Table 5. Model 4, comprising 9 items and a single factor, demonstrated the most adequate fit. The standardized factor coefficients, in conjunction with the fit indices, were deemed to be satisfactory: χ^22^(gl) 27.436 (24), CMIN/DF 1.143, CFI 0.96, GFI 0.97, SRMR 0.036, RMSEA 0.044. The final model is presented in Fig. 1.

**Table 5.** Fit indices obtained for each one of the tested models

Model	χ^2^ (gl)	CMIN/DF	CFI	GFI	SRMR	RMSEA
1	59.032	2.186	0.782	0.855	0.054	0.127
2	45.976	1.768	0.864	0.881	0.046	0.102
3	34.683	1.387	0.934	0.911	0.041	0.072
4	27.436	1.143	0.977	0.927	0.036	0.044
Cut-off criteria		<3	>0.95	>0.90	<0.05	<0.08

χ2 (gl): Chi-square (degrees of freedom); CMIN/DF: Chi-square ratio over degrees of freedom; CFI: comparative fit index; GFI: goodness-of-fit index; SRMR: standardized root mean square residual; RMSEA: root mean square error of approximation per degrees of freedom.

### Measurement invariance

A multi-group confirmatory factor analysis (CFA) was conducted for invasive mechanical ventilation (see Table 6). Initially, the configuration invariance model, baseline or free (M1), which proposed that the IPAT had a unifactorial structure in all groups and allowed factor loadings, intercepts, and error variances to be freely estimated, was tested. The indices obtained (CFI = .961; RMSEA = .037; χ^2^/gl = 1.098) indicated that the model demonstrated an adequate fit to the data (see Table 6). The metric invariance model (M2) was then tested, in which the factor loadings were restricted to be equal between individuals who did and did not require invasive mechanical ventilation.

**Table 6.** Results of tests of measurement invariance by invasive mechanical ventilation

Model	χ^2^(gl)	CMIN/DF	CFI	RMSEA (CI 90%)	Model comparison	Δχ^2^	Δ CFI	Δ RMSEA
M1. Configural invariance	70.277 (64)	1.098	0.961	0.037 (0.000−0.081)				
M2. Metric invariance	83.169 (73)	1.139	0.937	0.044 (0.000–0.083)	M2 vs M1	12.892 (9) *P* = 0.168	−0.007	0.007
M3. Scalar invariance	103.689 (83)	1.249	0.871	0.058 (0.000–0.092)	M3 vs M2	20.519 (10) *P* = 0.025	−0.01	0.014
M4. Strict invariance	145.046(97)	1.498	0.700	.082 (0.053–0.109)	M4 vs M3	38.741 (13) *P* = 0.000	0.024	0.024

The indices demonstrated that the model exhibited a high degree of fit, with a ΔCFI < 0.01, a ΔRMSEA < 0.015, and a non-significant Δχ^2^ (*P* < 0.05). The scalar invariance model (M3), in which the intercepts, in addition to the factor loadings, were restricted to equal between groups (by invasive mechanical ventilation), demonstrated an adequate fit. When compared to M2, there were no significant changes in CFI and RMSEA, nor in χ^2^. Conversely, the strict invariance model (M4), which incorporates the restriction of error variances in addition to factor loadings and intercepts, demonstrated an inadequate fit. In comparison with M3, the ΔCFI and ΔRMSEA both > 0.01, and the Δχ^2^ was found to be significant.

The same analysis was performed for the gender variable (males and females) (see Table 7), and the strict invariance model (M4), proved to be a more appropriate model than M3. The CFI was < 0.01 and the RMSEA was < 0.015, and the difference between the χ^2^ was not significant.

**Table 7.** Results of tests of measurement invariance by gender

Model	X^2^(gl)	CMIN/DF	CFI	RMSEA (CI 90%)	Model comparison	Δχ^2^	Δ CFI	Δ RMSEA
M1. Configural invariance	55.183 (6)	1.150	0.954	0.046				
				(0.000−.093)				
M2. Metric invariance	62.377 (52)	1.114	0.959	0.040	M2 vs M1	7.194 (8) *P* =0.516	0.005	−0.006
				(0.000–0.086)				
M3. Scalar invariance	78.007 (43)	1.200	0.916	0.053	M3 vs M2	15.630 (9) *P* = 0.075	−0.043	0.013
				(0.005–.092)				
M4. Strict invariance	101.357 (30)	1.299	.849	0.068	M4 vs M3	23.302 (12) *P* = 0.025	−0.067	0.015
				(0.030–0.097)				

### Criteria validity

A Pearson correlation (Table 8) was performed to determine the validity convergent and discriminant of the IPAT, with the subscales the EORTC QLQ C30. While no significant negative correlation was observed with global quality of life, a significant negative correlation was identified with emotional (−0.709, *P* < 0.001), cognitive (−0.413, *P* < 0.001), and fatigue symptoms (0.478, *P* < 0.001). 478, *P* < 0.001), dyspnea (0.458, *P* < 0.001), insomnia (0.265, *P* < 0.021), and finances (0. 242, *P* < 0.037), which is similarly addressed within the IPAT.

**Table 8.** Criteria validity (IPAT and EORTC-QLQ-C30 subscales)

	IPAT
**Functional scales**	
Physical functioning	−0.166
Role functioning	−0.155
Emotional functioning	−0.709**
Cognitive functioning	−0.413**
Social functioning	−0.134
**Symptoms cales/items**	
Fatigue	0.478**
Nausea and vomiting	0.156
Pain	0.055
Dyspnea	0.458**
Insomnia	0.265*
Appetite loss	0.180
Diarrhea	0.048
Constipation	0.055
Financial difficulties	0.242*
QoL	−0.131

QoL = Quality of Life,

** *P* < 0.01, *P 0.05.

Moreover, a known-groups validity (Davidson 2014) analysis was conducted to examine whether the IPAT scores could discriminate between patients based on clinical variables, it was supported by small to moderate SMD (see Table 9). The largest difference was observed between patients undergoing active cancer treatment and those who were not (SMD = 0.70).

**Table 9.** Known-groups validity for IPAT scores, based on clinical variables

Variable	Group Comparison	M (SD) – G1	M (SD) – G2	Mann–Whitney U	*P*	SMD	Effect size
Comorbidity	Yes vs No	15.32 (3.20)	13.86 (4.36)	446.500	0.043	0.38	Small
Invasive Mechanical Ventilation	Yes vs No	15.11 (3.16)	13.70 (4.60)	487.500	0.068	0.36	Small
Active cancer treatment	Yes vs No	13.78 (3.71)	16.78 (4.64)	256.000	0.010	0.70	Moderate

SMD= Standardized media difference; SD= Standard Deviation; M= Mean; U-tests for dichotomous variables. Significance level *P* < 0.05; Effect size: 0.20 = small; 0.50 = medium, 0.80 or above = large.

## Discussion

The aim of this study was to determine the psychometric properties of the IPAT for Mexican cancer population.

The IPAT was developed as a rapid and simple screening tool for routine use to detect psychological distress in patients admitted to the ICU. This problem is well-documented in ICU (Samuelson 2007; Wade et al. 2012, 2014; Davydow et al. 2009); was developed in accordance with the requirements of the UK NICE guideline CG83, which stipulates that all individuals admitted to the ICUs should undergo a psychological assessment, with the objective of providing patients with psychological support to detect and reduce psychological distress (Tan et al. 2009).

However, no validated and easy-to-use screening tool was available for this purpose in Mexican cancer population.

It is essential to address this public health issue, because it has been identified that cancer patients who require admission to the ICU in Mexico present significant psychological alterations, because of both the emotional impact of their cancer diagnosis and the critical experience lived in the ICU (Ascencio-Huertas et al. 2021). Despite this double vulnerability, these alterations have not been documented systematically or in a timely manner in the national literature, which has contributed to the invisibility of post-ICU distress suffering in this population and to the increase in its negative impact on quality of life.

In this context, valid, reliable and culturally adapted assessment tools are indispensable for health professionals to be able to identify emerging psychological symptoms early and intervene in a timely manner. Cultural differences can profoundly influence the way people understand and respond to items on a scale (Ramada-Rodilla et al. 2013), especially in highly emotionally charged contexts such as ICU hospitalization.

The validation study, conducted with 75 critically ill cancer patients in Mexico, confirmed that nine of the original 10 items formed a reliable feasible and acceptable unitary scale measuring psychological distress due to intensive care.

Subsequent analysis indicated that the IPAT factor structure was highly analogous to the original version, demonstrating adequate internal consistency, content validity, construct validity, and criterion validity.

The results supported the dimensionality proposed by the IPAT, with satisfactory fit indices by keeping the factor structure constant according to gender and invasive mechanical ventilation, except for one parameter of the strict invariance model. Partial invariance was assumed (Dimitrov 2010), as these tests are often too restrictive (Bentler 2004). Even so, scores remained predominantly comparable across groups, with equivalent meaning for one-unit changes.

The IPAT has been demonstrated to function as a clinical screening tool for detecting psychological distress in ICU patients who are awake, alert, and oriented. Healthcare professionals must be trained to identify and refer ICU patients with psychological distress for timely support, addressing their mental health needs during hospitalization and beyond. Conversely, although a mixed analysis was not conducted to integrate qualitative results, an additional open-ended item was incorporated into the tool to enable subjects evaluated to disclose any other adverse psychological experiences they encountered during their stay in the ICU.

This item facilitated comprehension of the context and specific needs of the population group in question. While extant literature highlights concern regarding the adverse mental health consequences of ICU stays and admission, concerns regarding the family and socioeconomic consequences of such an experience are also emphasized in this population (Cedeño-Vivar et al. 2021; Gómez-Carretero et al. 2007; Morales-Rolo 2020).

Lack of financial resources, as well as loss of independence and no paid activities, represent major issues that are viewed as a concern compared to their stay in this unit for cancer, which in turn negatively impacts their mental health after the ICU (Barragán-Becerra et al. 2018; Coca and Fernández 2013).

In a similar context, female patients who identified as mothers indicated that their foremost concern was the care and attention of their children, a priority that superseded their own clinical condition due to cancer and subsequent admission to the ICU. It is notable that this issue is most frequently reported by women, necessitating consideration of the psychosocial and gender context of clinical care for this population. This underlines the importance of considering gender differences in healthcare to take timely action and raise awareness of these issues, highlighting the need to address the historical, political and social determinants that affect mothers (Asensio 2025; López-Ferreruela et al. 2024; Mirelman and Extremera 2025).

This indicates that the psychological assessment routinely conducted on patients admitted to ICU should not be limited to the scores on the clinical scales used but should also allow for an in-depth examination of the specific needs of each person who requires it (Gómez-Carretero et al. 2007; Pardavila-Belio and Vivar 2012; Valdiviezo-Verdezoto et al. 2024).

In this regard, the results show clinically and statistically significant evidence of known group validity for the IPAT. Specifically, higher levels of psychological distress were observed in people with more clinically vulnerable conditions, among those receiving oncological treatment, those receiving invasive mechanical ventilation in ICU and those with comorbidity. This suggests that the IPAT may be sensitive to certain following ICU risk conditions, although a larger sample size is recommended to strengthen the statistical power of the analysis.

Also, this study has several limitations that should be acknowledged. First, the research was conducted in a single oncology referral center in Mexico, which may limit the generalizability of the findings to broader or more diverse populations (Polit and Beck 2021). Cultural, institutional, and healthcare system differences could influence patient experiences and the interpretation of psychological constructs, even within the same language group.

Additionally, the study included only native Spanish speakers, excluding indigenous language speakers who often face healthcare inequities, language barriers, and culturally influenced health behaviors and psychological responses.

This can result in a form of epistemic injustice, where certain groups of psychological experiences are rendered invisible or misunderstood because appropriate tools are not available for their assessment (Harper and Thompson, 2011). So, representativeness of the sample highlights the need for future validation and adaptation efforts in linguistically diverse subpopulations (Gutiérrez et al. 2019).

Second, despite meeting a minimum functional level (Karnofsky ≥ 50), recent ICU discharge and during their hospitalization after ICU may have affected patients’ cognitive or emotional capacity to respond accurately, as following ICU impairments like memory and attention issues (Girard et al. 2010; Pandharipande et al. 2013), which could impact self-report reliability.

Regarding criteria validity, carried out with the EORTC QLQ C30, which reports alphas of less than 0.70, standing out in the cognitive factor (α = 0.32), the IPAT obtained a moderate and significant negative correlation (−0.413 *P* < 0.01); however, due to its low indices it is advisable to interpret with caution and consider them in greater depth in future research.

Finally, social desirability bias may have played a role, as assessments were conducted verbally and in a hospital setting, which could influence participants’ willingness to disclose distressing psychological symptoms.

Despite these limitations, this study highlights the need to expand research on psychological distress in cancer patients following ICU discharge. Future studies should include multicenter samples across different Mexican regions to enhance generalizability and explore regional or institutional differences. It is also necessary to validate assessment tools in subpopulations with diverse sociodemographic profiles (such as gender, race, class, age, language, etc.) whose psychological experiences and access to care may differ significantly.

Incorporating an intersectional perspective will allow for a better understanding of how structural and social determinants influence psychological vulnerability and response to critical illness because of cancer (Bowlegn 2012).

Longitudinal research is also needed to monitor how distress evolves after ICU discharge and its long-term impact on quality of life, due to hospitalization time following ICU and how the assessment during this stage may reflect different data afterwards.

In conclusion, the IPAT is a valid and reliable instrument with adequate psychometric properties and adjusted indices to detect psychological distress in ICU patients with cancer in Mexico. Based on the current results, the IPAT, following replication and further validation, could serve as a tool for ICU staff to identify psychological distress in patients. In addition, its use will allow comparison of results nationally and internationally. It is suggested that future research on the IPAT examine the proposed factor structure, considering, in the case of our population, the socioeconomic and family context faced by each cancer patient admitted to the ICU, which defines specific needs in each of them.

## Supporting information

10.1017/S1478951525100667.sm001 Flores-Constantino et al. supplementary material Flores-Constantino et al. supplementary material

## Supplementary material

The supplementary material for this article can be found at https://doi.org/10.1017/S1478951525100667.
