# Supplementary material for: Assessing psychological distress in cancer patients in intensive care: Validation of the intensive care psychological assessment tool in Mexico
Source: Palliat Support Care. 2025 Aug 28;23:e152. doi: 10.1017/S1478951525100667 (PMC13166314; doi:10.1017/S1478951525100667)
Supplement: Flores-Constantino et al. supplementary material [file S1478951525100667sup001.docx]

**IPAT translation and back translation process in Mexico for cancer patients***

First, to achieve semantic equivalence of the instrument, it was necessary to translate it from its original British English into Mexican Spanish and analyze the translation in detail. Subsequently, this Mexican Spanish version was translated back into the original language and checked for consistency with the original IPAT (Gómez and Ospina, 2001; Wild et al., 2005):

1. For the direct translation, two independent translations of the original version (British English) into the language of the target population (Mexican Spanish) were carried out by professional translators with knowledge of the two cultures and the construct. The two translators had to communicate fluently in both languages but were asked to translate into their native language (in this case, Mexican Spanish) and to know or be immersed in their culture, so that they could use colloquial expressions typical of the target population. They were made aware of the intention of each item and of the scale, with the aim of respecting the original content of each item, trying to reflect the meaning of the construct, but using expressions specific to the target population (Reyes Lagunes and García-Barragán, 2008).
2. Once direct translation was available, in coordination with the translators and the authors of the present study, the decision was made to choose the most appropriate version, considering a) that the meaning and intention of the item was preserved and b) that the language was clear and natural for the target population.
3. After the best translated version was decided by consensus, it was sent for back-translation into the original language in British English by other independent bilingual translators, whose native language on this occasion was that of the original version. In this step, the translators were not supposed to know the scale and its purpose (Muñiz, Elosua and Hambleton, 2013).
4. When the back translated version was available, it was analyzed item by item for comparison with the original version, the degree of concordance was identified based on the assessments of the translators of both languages, as well as the experts of the construct, and the best form of expression for each item was decided for the target population.
5. In case of significant discrepancies, two independent experts would be asked to assess the semantic equivalence of each of the items obtained in the previous step, on a six-point scale: from Totally different or altered (1) to Totally equivalent or unchanged (6). Items evaluated with less than five points had to be translated and re-translated again until this score was achieved (García Cadena, 2009), however, according to the versions obtained, no item had considerable discrepancies, including instructions and response options, which were integrated in each of the steps mentioned above.

| **DIRECT AND BACK TRANSLATION OF THE ITEMS AND FINAL VERSION: IPAT** | | | |
| --- | --- | --- | --- |
| *Original English version* | *Direct translation*  *(English to Spanish)*  *Translator A (TrA)*  *Translator B (TrB)*  *Only the best translation of each item (marked in bold) is sent for re-translation.* | *Back translation*  *(Spanish to English)*  *Translator C (TrC)*  *Translator D (TrD)* | *Final version in Spanish* |
| Instructions:  I would like to ask you some questions about your stay in intensive care, and how you’ve been feeling in yourself, These feelings can be an important part of your recovery. To answer, please circle the answer that is closest to how you feel, or answer in any way you are able to (e.g. by speaking or pointing.) | TrA: Me gustaría hacerle algunas preguntas sobre su estancia en cuidados intensivos y cómo se ha sentido consigo mismo(a). Estos sentimientos pueden ser una parte importante de su recuperación. Para responder, encierre en un círculo la respuesta que más se acerque a cómo se siente, o responda de cualquier manera que pueda (por ejemplo, hablando o señalando). |  | Instrucciones:  Me gustaría hacerle algunas preguntas sobre su estado emocional respecto a su estancia en cuidados intensivos, lo cual es una parte importante de su recuperación. Para contestar, coloque una **X** sobre la respuesta que explique mejor cómo se siente, o puede responder de la manera que le sea posible (por ejemplo, hablando o señalando). |
|  | TrB: **Me gustaría hacerle algunas preguntas acerca de su estancia en Cuidados Intensivos, sobre cómo se ha sentido al respecto. Sus sentimientos y emociones pueden ser una parte importante de su recuperación. Para responder, por favor circule la respuesta que se acerque más a cómo usted se siente o responda de la mejor manera que le sea posible (p. ej. Hablando o señalando)** | **TrC**: I would like to ask you a few questions about your stay in the Intensive Care Unit and how you have been feeling about yourself. These feelings can play an important role in your recovery. Please respond by circling the answer that best reflects how you feel, or by responding in any way you are able to (for example, by speaking or pointing). |  |
|  |  | **TrD:** I would like to ask you some questions about your stay at intensive care and how you have been feeling about yourself. These feelings may be an important part of your recovery. To answer, circle the answer that comes closest to how you feel, or answer in any way you can (for example, by talking or pointing) |  |
| Has it been hard to communicate? | TrA: ¿Ha sido difícil comunicarse? |  | ¿Le ha sido difícil comunicarse? |
|  | TrB: **¿Le ha sido difícil comunicarse?** | **TrC:** Have you experienced any difficulty with communication? |  |
|  |  | **TrD:** Has it been difficult to communicate? |  |
| Has it been difficult to sleep? | TrA: ¿Ha sido difícil dormir? |  | ¿Le ha sido difícil dormir? |
|  | TrB: **¿Le ha sido difícil dormir?** | **TrC:** Have you had trouble sleeping? |  |
|  |  | **TrD:** Has it been difficult to sleep? |  |
| Have you been feeling tense? | TrB: ¿Se ha sentido tenso? |  | ¿Se ha sentido tenso/a? |
|  | TrA: ¿Se ha sentido tenso/a? | **TrC:** Have you felt tense or anxious? |  |
|  |  | **TrD:** Have you felt tense? |  |
| Have you been feeling sad? | TrA: **¿Se ha sentido triste?** | **TrC:** Have you experienced feelings of sadness? | ¿Se ha sentido triste? |
|  |  | **TrD:** Have you felt sad? |  |
|  | TrB: **¿Se ha sentido triste?** |  |  |
| Have you been feeling panicky? | TrA: ¿Ha sentido pánico? |  | ¿Ha sentido pánico? |
|  | TrB: **¿Ha sentido pánico?** | **TrC:** Have you felt panic or intense fear? |  |
|  |  | **TrD:** Have you felt panicky? |  |
| Have you been feeling hopeless? | TrA: **¿Se ha sentido sin esperanza?** |  | ¿Se ha sentido sin esperanza? |
|  | TrB: **¿Se ha sentido sin esperanza?** | **TrC:** Have you experienced feelings of hopelessness? |  |
|  |  | **TrD:** Have you felt hopeless? |  |
| Have you felt disorientated (not quite sure where you are)? | TrB: **¿Se ha sentido desorientado/a (no muy seguro/a de dónde se encuentra)?** |  | ¿Se ha sentido desorientado/a (no muy seguro/a de dónde se encuentra)? |
|  | TrA: **¿Se ha sentido desorientado/a (no muy seguro/a de donde se encuentra?** | **TrC:** Have you felt disoriented or unsure of your surroundings? |  |
|  |  | **TrD:** Have you felt disoriented (not quite sure where you are)? |  |
| Have you had hallucinations (seen or heard things you suspect were not really there)? | TrB: ¿Ha tenido alucinaciones (ver o escuchar cosas que sospechaba que en realidad no existían)? |  | ¿Ha tenido alucinaciones (escuchar o ver cosas que sospechaba que no existían)? |
|  | TrA: **¿Ha tenido alucinaciones (escuchó o vio cosas que sospechaba que en realidad no existían)?** | **TrC:** Have you experienced hallucinations (e.g., hearing or seeing things you suspected were not real)? |  |
|  |  | **TrD:** Have you had hallucinations (heard or seen things you suspected were not really there)? |  |
| Have you felt that people were deliberately trying to harme or hurt you? | TrA: ¿Ha sentido que las personas estaban tratando deliberadamente de lastimarle o herirle? |  | ¿Ha sentido que las personas estaban tratando de lastimarle o herirle a propósito? |
|  | TrB: **¿Ha sentido que alguien ha tratado de lastimarle o herirle a propósito?** | **TrC:** Have you felt that others were intentionally trying to harm or hurt you? |  |
|  |  | **TrD:** Have you felt that people were deliberately trying to hurt or harm you? |  |
| Do upseting memories of intensive care keep coming into your mind? | TrB: ¿Siguen viniendo a su mente recuerdos angustiantes de cuidados intensivos? |  | ¿Vienen a su mente recuerdos angustiantes o desagradables de su estancia en Cuidados Intensivos? |
|  | TrA: **¿Tiene recuerdos angustiantes o desagradables de su estancia en cuidados intensivos que se mantienen en su memoria?** | **TrC:** Have distressing or unpleasant memories of your stay in the Intensive Care Unit come to mind? |  |
|  |  | **TrD:** Do you have distressing memories of your time in intensive care that remain in your memory? |  |
| *Response options*  No  Yes, a bit  Yes, a lot | TrA: Nada  Si, un poco  Si, mucho |  | *Opciones de respuesta*  Nada  Un poco  Mucho |
|  | TrA: No  Si, un poco  Si, mucho |  |  |

*References included in the article
